# Low-dose naltrexone and NAD+ for the treatment of patients with persistent fatigue symptoms after COVID-19

**DOI:** 10.1016/j.bbih.2024.100733

**Published:** 2024-02-01

**Authors:** Anar Isman, Andy Nyquist, Bailey Strecker, Girish Harinath, Virginia Lee, Xingyu Zhang, Sajad Zalzala

**Affiliations:** a AgelessRx, 2370 E Stadium Blvd #2049, Ann Arbor, MI, 48104, USA; b Thomas E. Starzl Transplantation Institute, University of Pittsburgh, Pittsburgh, PA, 15213, USA

**Keywords:** COVID-19, Long COVID, Fatigue, Quality of life, Low dose naltrexone, NAD+, SF-36

## Abstract

A subset of patients experiences persistent fatigue symptoms after COVID-19, and patients may develop long COVID, which is characterized by lasting systemic symptoms. No treatments for this condition have been validated and are urgently warranted. In this pilot study, we assessed whether treatment with low-dose naltrexone (LDN, 4.5 mg/day) and supplementation with NAD + through iontophoresis patches could improve fatigue symptoms and quality of life in 36 patients with persistent moderate/severe fatigue after COVID-19. We detected a significant increase from baseline in SF-36 survey scores after 12 weeks of treatment (mean total SF-36 score 36.5 [SD: 15.6] vs. 52.1 [24.8]; p < 0.0001), suggestive of improvement of quality of life. Furthermore, participants scored significantly lower on the Chalder fatigue scale after 12 weeks of treatment (baseline: 25.9 [4.6], 12 weeks: 17.4 [9.7]; p < 0.0001). We found a subset of 52 % of patients to be responders after 12 weeks of treatment. Treatment was generally safe, with mild adverse events previously reported for LDN, which could be managed with dose adjustments. The iontophoresis patches were associated with mild, short-lived skin irritation in 25 % of patients. Our data suggest treatment with LDN and NAD+ is safe and may be beneficial in a subset of patients with persistent fatigue after COVID-19. Larger randomized controlled trials will have to confirm our data and determine which patient subpopulations might benefit most from this strategy.

## Highlights

• A subset of patients experienced persistent fatigue symptoms after COVID-19.
• Treatment with low-dose naltrexone (LDN) and NAD+ was well tolerated.
• Treatment increased SF-36 quality of life scores.
• Treatment also improved fatigue symptom scores.
• A subset of patients were clinically responsive.

## 1 Introduction

Acute infection with severe acute respiratory syndrome coronavirus 2 (SARS-CoV-2) results in coronavirus disease 2019 (COVID-19). While most patients with mild diseases recover within a couple of weeks, even after mild disease patients may develop long COVID (or post-acute COVID-19), characterized by lasting systemic symptoms. Definitions of long COVID vary and are expected to continue to evolve with further research into this condition. The World Health Organization (WHO) defines long COVID as the continuation or development of new symptoms three months after initial SARS-CoV-2 infection, with these symptoms lasting for at least two months with no other explanation, while the Centers for Disease Control and Prevention in the US (CDC) use a broader definition of signs, symptoms, and conditions that continue or develop after initial COVID-19 infection. Various global studies have attempted to estimate the incidence of long COVID, but due to a lack of clear diagnostic criteria, variability in registration, and differences in timing of measurements, reported estimates vary between 10 and 51 % (Altay et al., 2021; Bureau NCfHSUC.,2023; Chen et al., 2022; O'Mahoney et al., 2023). Furthermore, recent studies have shown that the risk of persistence of symptoms after acute infection might be decreasing over time, potentially linked to vaccination and different SARS-CoV-2 variants, even though reinfections have been associated with a higher rate of post-acute sequelae than first infections. Davis et al. estimated that at least 65 million people worldwide may have long COVID, based on an estimated incidence of 10 % of infections resulting in long COVID, but given the high differences in estimates, this number might be much higher (Ballering et al., 2022; Davis et al., 2023).

Long COVID has been associated with a wide array of symptoms, including cough, dyspnea, chest pain, palpitations, abdominal pain, nausea, cognitive impairment, persistent and severe fatigue, neurocognitive dysfunction including brain fog, tinnitus, post-exertional malaise, orthostatic intolerance, and sleep disruptions (reviewed in Davis et al., 2023) (Davis et al., 2023)). Furthermore, long-term consequences of acute infections and long COVID have been reported as risk factors for various diagnoses including cardiovascular disease (Luchian et al., 2023), postural orthostatic tachycardia syndrome (POTS) (Luchian et al., 2023), and new-onset diabetes (Harding et al., 2023). Additionally, many long COVID symptoms overlap with symptoms of ME/CFS, suggesting the clinical presentation of these conditions to be similar (Komaroff and Lipkin, 2023; Wong and Weitzer, 2021). Therefore, many patients meet an ME/CFS case definition (Jason and Dorri, 2022). It remains unclear what percentage of patients with long COVID recover spontaneously over time. One study that followed a cohort of 968 patients over time found that among those with symptoms two months after initial infection, 85 % had persistent symptoms one year after onset. Three patterns were observed when assessing 53 symptoms; 27 symptoms decreased in prevalence, including loss of taste/smell and cough, 8 symptoms increased in prevalence, including paresthesia, and 18 symptoms did not change much over time, including dyspnea (Tran et al., 2022). The symptoms started to have the most impact on the quality of life of patients after six months (Tran et al., 2022).

Currently, no treatments have been validated for the post-acute symptoms of COVID-19, though research has suggested some targets for therapy.

One such strategy is naltrexone, a non-selective opioid antagonist, with a high affinity for μ-opioid receptors. It is FDA-approved for the medication-assisted treatment of opioid and alcohol dependence. Standard doses for these conditions are 50–150 mg/day, which also prevents the inhibition of the gamma-aminobutyric acid receptor and inhibits dopamine release. In doses below 5 mg (LDN), naltrexone acts as a glial modulator and does not fully block opioid receptor signaling. Its blockade lasts 4–6 h, during which an increased endogenous opioid production and production of opioid receptors is initiated. The increased production of endogenous opioids also modulates the immune system by inhibiting the proliferation of B and T cells (Li et al., 2018). LDN also blocks the opioid growth factor (OGF) receptor, which leads to a feedback loop resulting in increased production of endogenous OGF and the OGF receptor, increasing signaling. LDN may also bind directly to the OGF receptor on immune cells, thereby functioning as an immune modulator (Patten et al., 2018). Binding of OGF to the OGF receptor and increased endogenous opioid signaling can play a role in supporting the growth and development of tissues and organs. Therefore, LDN may promote cell proliferation, wound healing, and reduce inflammation. LDN is also a specific antagonist for Toll-like receptor 4 (TLR4) on immune cells, inhibiting the downstream signaling pathways responsible for the production of pro-inflammatory cytokines (Trofimovitch and Baumrucker, 2019). One of the cytokines LDN reduces is interleukin-6 (IL-6) (Parkitny and Younger, 2017), which is upregulated during acute infection and in patients with long COVID, and the highest levels are detected in those with severe disease (Potere et al., 2021; Patterson et al., 2021). Given its role in modulating the immune response, LDN may aid the body in the viral clearance of SARS-CoV-2 and may aid in preventing immunothrombosis caused by infection (Choubey et al., 2022; Pitt et al., 2022).

LDN has been widely used off-label for the treatment of inflammation and pain in autoimmune diseases, such as multiple sclerosis (MS), Crohn's disease, and fibromyalgia (Patten et al., 2018; Toljan and Vrooman, 2018). LDN has also been used off-label for ME/CFS, despite a lack of clinical trials. A case series describing three cases with ME/CFS treated with LDN showed that responses vary, potentially due to dosing, with patients self-reporting improved energy, sleep, mood, and pain levels (Bolton et al., 2020). Furthermore, in a retrospective study in Finland including 218 patients with ME/CFS who received LDN, 73.9 % of patients self-reported a positive response, including physical and mental effects, while 18.3 % did not report any treatment effects. Adverse events were considered mild and more common at the beginning of treatment (Polo et al., 2019/10). In a previous study among 38 individuals with post-COVID sequelae who were treated with LDN at 1–3 mg/day, improvements in various self-reported measures were detected after two months of therapy. These included limitations in activities of daily living, energy, pain, concentration, sleep disturbance, and recovery from COVID-19, with the largest improvements seen in pain-related measures (O'Kelly et al., 2022). Furthermore, before the onset of this study, patients reached out to our clinic noting that LDN was helpful for their post-COVID symptoms. These data suggest LDN may be beneficial in this patient population. However, the discussed retrospective studies did not include a control arm, did not make use of objective standardized tests, and doses of LDN varied among patients, making it challenging to draw overall conclusions for this patient population, and randomized controlled trials are needed.

Nicotinamide adenine dinucleotide (NAD) is a coenzyme involved in redox reactions. It is found in every cell of the human body and is involved in several metabolic pathways. It catalyzes electron transfer in metabolic reduction-oxidation reactions, essential for ATP production. In aging and obese populations, NAD + has been found to decline over time (Sultani et al., 2017). SARS-CoV-2 infection in human and animal cells has been shown to result in the upregulation of poly-ADP ribose polymerase (PARP) family member genes, major consumers of NAD, resulting in a depletion of NAD + levels (Heer et al., 2020). SARS-CoV-2 infection has also been suggested to increase activity of CD38 on immune cells, also depleting NAD levels (Horenstein et al., 2021). NAD + deficiency reduces the function and activation of SIRT1, a NAD-dependent deacetylase that is involved in various physiological functions, including aging, metabolism, and regulation of gene expression. SIRT1 also downregulates the transmembrane protease ADAM17, which aids in decreasing the cytokine levels of TNFα, Il-1β, and Il-6. Therefore, if ADAM17 expression is not downregulated by SIRT1 during an infection, it may lead to uncontrolled cytokine production as occurring in some COVID-19 patients (Miller et al.,2020). Reduction of NAD + levels and associated mechanisms may be related to the immune response seen in COVID-19 and it has been suggested that supplementing NAD+ in the elderly population and those with comorbidities may aid in reducing the severe and long haul effects of COVID-19 (Miller et al., 2020; Omran and Almaliki, 2020).

We hypothesized that treatment with LDN and NAD + supplementation may aid in reducing symptoms of moderate/severe fatigue in the chronic phase of COVID-19. We conducted an observational open-label pilot study in which patients who had persistent fatigue symptoms were treated for 12 weeks and assessed for changes in the quality of life and fatigue using the SF-36 and Chalder scale surveys.

## 2 Methods

### 2.1 Study design

We conducted an observational open-label pilot study to address the chronic phase of COVID-19 (NCT04604704) assessing the effects of LDN and NAD + treatment on reducing fatigue and improving quality of life. The study was conducted in accordance with the principles in the Declaration of Helsinki and the study protocol was approved by the institutional review board of the Institute of Regenerative and Cellular Medicine (IRCM) (approval no. IRCM-2020-265). The study was initially set up as a placebo-controlled trial, but was converted into an interventional single arm study due to challenges with participant accrual.

### 2.2 Participants

Patients aged between 18 and 65 years were eligible if they had a positive SARS-CoV-2 PCR, antigen, or antibody test 1–12 months before enrollment. At enrollment, the Chalder Fatigue Scale was used to determine eligibility, with scores over 9 (moderate to severe fatigue) considered eligible for enrolment. Patients with clinically significant renal, cardiovascular, or hepatic impairment, those taking opioid analgesics or undergoing treatment for opioid addiction, with a known sensitivity to naltrexone, a suspected or confirmed pregnancy or while breastfeeding, known issues with the use of iontophoresis patches, active cancer, enrolled in another trial, or current users of LDN or NAD+ were excluded. All patients provided informed consent for participation in this study.

### 2.3 Treatment

Participants were treated for 12 weeks with LDN and NAD+. Participants were actively seeking out treatment for their fatigue symptoms and were found to be largely unwilling to participate in a placebo-controlled trial, with the risk of not receiving treatment. This resulted in difficulties in the accrual of enough patients for the trial, and therefore, after July 30, 2021, patients were placed in an unblinded open-label observational treatment arm, and we offered participants (N = 3) initially randomized into the control group to enroll in the treatment group. Participants were enrolled via a virtual telemedicine platform and received trial prescriptions by mail. LDN was prescribed at a dosage of 4.5 mg/day taken in the evening before bed, LDN tablets were custom compounded (Pharmacy Solutions, Ann Arbor, MI or Belmar Pharmacy, Golden, CO). The LDN dose was slowly built up in nine days, with a quarter of the dose for the first four days, half a dose for the next four days, and the final full dose from day 9 onward. Since oral supplementation of NAD has limited bioavailability, and self-injections pose challenges for a pilot trial, NAD+ was applied using iontophoresis patches (IontoPATCH™️ STAT, ST. Paul, MN; provided by Pharmacy Solutions, Ann Arbor, MI or Belmar Pharmacy, Golden, CO). The patches consisted of ready-made 400 mg/mL NAD + solution, with 1 mL that was applied to the positive electrode of the patch and saline applied to the negative electrode. Patches were worn for 4–6 h, once a week. If participants experienced rash at the patch application site, they were offered a prescription for a steroid cream for the area and were instructed to move the administration area to allow any skin irritation to heal.

### 2.4 Assessments

Validated surveys were completed by participants at baseline, and at 2, 4, 8, and 12 weeks after the start of treatment. The Chalder Fatigue Scale is a widely used self-assessment tool for chronic fatigue, including ME/CFS (Jackson, 2015). This scale was used to assess fatigue symptoms, and the Short Form 36 (SF-36) survey was used to measure various aspects of quality of life. The SF-36 is a validated survey measuring the quality of life of healthy adults or patients (Ware et al.,1992, and Sherbourne; Hayes et al., 1995; Jenkinson et al., 1993). The Chalder Fatigue Scale was assessed using Likert scaling. Furthermore, participants were asked to answer surveys assessing adverse events, and other health-related questions.

### 2.5 Statistical analysis

The Safety dataset included all participants who received at least one dose of the study intervention, while the Efficacy dataset included all participants who had received at least one dose of the study intervention and completed at least baseline and week 2 survey assessments. Mean and standard deviations of health items from various surveys were calculated with a mixed linear model for repeated measures. The SF-36 and Chalder survey scores were analyzed using the Chi-square test or the Fisher's exact test. Clinically responsive participants were defined as having improved >20 % from baseline and having a Week 12 value > 55 for SF-36 scores. Clinically unresponsive was defined as those with a Week 12 value < 55 for SF-36 scores. Analyses were performed using R version 4.0.2. All tests were performed two-sided and P-values <0.05 were considered statistically significant.

## 3 Results

### 3.1 Demographics

This interventional pilot study was conducted between March 31, 2021, and December 22, 2022. We included a total of 36 participants in the intervention group (Safety dataset), of whom 5 withdrew from the study, and no efficacy data were obtained. Therefore, 31 participants were included in the Efficacy dataset (Supplementary Fig. 1). Participants included in the Safety dataset tested positive for SARS-CoV-2 infection between August 27, 2020, and August 11, 2022, and the majority of infections were confirmed with a PCR test (86.1 %). Ages ranged from 28 to 69 years, and 69.4 % of participants were female (Table 1). The time between a positive COVID-19 test and the first study dose varied from 27 to 624 days, with a mean of 223.29 days.

**Table 1:** Demographics and participant characteristics.

	Safety Dataset (N = 36)
**Age** (years)	
Mean (SD)	44. 7 (12.0)
Range	28–69

**Sex** (n ( %))	
Male	11 (30.6)
Female	25 (69.4)
**Time between COVID-19 test and first trial dose** (days)	
Median (IQR)	193.5 (122.5)
Range	27–624

**Type of COVID-19 test** (n ( %))	
PCR test	31 (86.1 %)
Antigen test	5 (13.9 %)

### 3.2 Safety

Self-reported adverse events are listed in Table 2. The most commonly reported adverse event was skin irritation in the location of the NAD + patch (n = 11). Participants were offered a topical cream to treat skin irritation, but only one participant accepted this treatment. All skin irritation was temporary and resolved within days after the removal of the patch. Nausea, fatigue, dizziness, and low mood occurred mainly during the first weeks of treatment and were managed with dosing schedule adjustments. Since these adverse events have been observed with LDN previously, these are likely attributable to the LDN treatment. Insomnia was managed by changing the treatment dosing to the morning for either LDN or NAD+. One participant reported hypotension and was diagnosed by their regular health care team with POTS, suggesting the hypotension is unlikely related to the study intervention. One participant reported pregnancy after the initiation of treatment and was advised to stop treatment. However, the participant had a miscarriage after a COVID-19 re-infection, which is likely unrelated to the study intervention, and continued treatment.

**Table 2:** Self-reported adverse events.

Adverse Event	Safety Dataset (N = 36)
	n ( %)
Skin irritation	11 (30.6)
Viral/bacterial infection	5 (13.9)
Fatigue	4 (11.1)
Dizziness	2 (5.6)
Insomnia	2 (5.6)
Low mood	3 (8.3)
Vomiting	1 (2.8)
Diarrhea	1 (2.8)
Stomach pain	1 (2.8)
Confusion	1 (2.8)
Shortness of breath	1 (2.8)
Headache	2 (5.6)
Miscarriage	1 (2.8)
Hypotension	1 (2.8)

Five participants withdrew from the study within the first two weeks of treatment, of whom two were lost to follow-up. Three participants withdrew due to pre-existing medical issues and/or adverse events that are known to be caused by LDN (nausea, vomiting, diarrhea, shortness of breath, insomnia, dizziness).

### 3.3 Fatigue (Chalder) and quality of life (SF-36) survey data

**Fig. 1:**
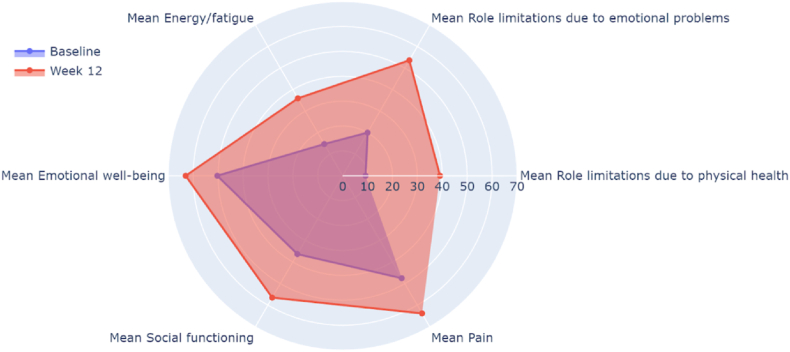
Radar Plot of SF-36 items from Baseline to Week 12.

Participants were asked to complete the SF-36 survey to assess quality of life at baseline, and following the start of the treatment at weeks 2, 4, 8, and 12. In the Efficacy dataset group (N = 31), the mean total SF-36 score improved from 36.5 (SD 15.6) at baseline to 42.0 (SD 18.9; p = 0.0060) at week 2, 42.9 (SD 21.1; P = 0.0008) at week 4, 50.0 (SD 23.0; P < 0.0001) at week 8, and 52.1 (SD 24.8; P < 0.0001) at week 12 (Table 3). A radar plot depicting improvements in various SF-36 categories from baseline to 12 weeks is depicted in Fig. 1. At week 12, all categories showed significant improvements when compared to baseline, but the effects were most pronounced for mean role limitations due to physical health (scores 9.2 [SD: 15.4] vs. 39.1 [39.8]), mean energy/fatigue (14.7 [11.3] vs. 35.9 [26.3]), and mean pain scores (47.5 [25.2] vs. 63.8 [29.9]) (Table 3).

**Table 3:** SF-36 and Chalder Fatigue Scale survey results.

	Mean (SD) of each item					p value of the difference vs. baseline			
	Baseline (N = 36)	Week 2 (N = 30)	Week 4 (N = 25)	Week 8 (N = 25)	Week 12 (N = 23)	Week 2 vs. baseline	Week 4 vs. baseline	Week 8 vs. baseline	Week 12 vs. baseline
**Mean Total SF-36**	36.5 (15.6)	42.0 (18.9)	42.9 (21.1)	50.0 (23.0)	52.1 (24.8)	**0.006**	**0.0008**	**<0.0001**	**<0.0001**
Mean Physical functioning	57.2 (27.6)	61.0 (28.3)	61.0 (29.1)	63.8 (30.8)	66.7 (29.3)	**0.0459**	**0.0171**	**0.0023**	**<0.0001**
Mean Role limitations due to physical health	9.2 (15.4)	22.5 (35.0)	21.0 (35.1)	29.0 (38.6)	39.1 (39.8)	**0.0198**	**0.0215**	**0.001**	**<0.0001**
Mean Role limitations due to emotional problems	20.0 (32.3)	25.6 (33.5)	37.3 (41.2)	60.0 (45.1)	53.6 (46.9)	0.5115	0.0517	**<0.0001**	**0.0002**
Mean Energy/fatigue	14.7 (11.3)	25.5 (17.4)	25.4 (18.8)	30.2 (22.1)	35.9 (26.3)	**0.0001**	**<0.0001**	**<0.0001**	**<0.0001**
Mean Emotional well-being	50.3 (16.6)	52.7 (16.0)	53.0 (20.3)	63.4 (18.9)	63.0 (20.5)	0.2044	0.2245	**0.0001**	**<0.0001**
Mean Social functioning	36.3 (26.1)	42.1 (24.9)	49.5 (29.2)	55.5 (27.3)	56.5 (30.8)	0.0696	**0.0005**	**<0.0001**	**<0.0001**
Mean Pain	47.5 (25.2)	55.2 (26.3)	54.2 (29.5)	63.5 (29.6)	63.8 (29.9)	**0.0057**	**0.0053**	**<0.0001**	**<0.0001**
Mean General Health	39.2 (21.6)	40.7 (22.5)	38.8 (23.2)	43.2 (23.5)	42.6 (23.8)	0.1422	0.2709	0.0888	**0.0125**
**Total Sum of Chalder Fatigue Scale**	25.9 (4.6)	21.3 (7.2)	19.9 (8.3)	18.5 (9.4)	17.4 (9.7)	**<0.0001**	**<0.0001**	**<0.0001**	**<0.0001**
Sum of Chalder Fatigue Scale - Physical	17.0 (3.1)	14.1 (4.5)	13.3 (5.2)	12.1 (6.3)	11.4 (6.0)	**0.0002**	**<0.0001**	**<0.0001**	**<0.0001**
Sum of Chalder Fatigue Scale - Mental	8.9 (2.1)	7.2 (3.1)	6.6 (3.4)	6.4 (3.6)	6.0 (4.0)	**0.0001**	**<0.0001**	**<0.0001**	**<0.0001**

SD: standard deviation; SF-36: short form 36.

An improvement in fatigue symptoms was observed with the Chalder survey results. The total sum of the scale decreased from 25.9 (SD: 4.6) at baseline to 17.4 (9.7) at week 12 (Table 3; p < 0.0001). For subsections of the Chalder scores, both physical and mental health improved significantly in the 12 weeks of follow-up.

We did not detect a correlation between the length of time between the positive COVID-19 test and the first date of the study drug and responsiveness, when participants were grouped as <6 months, ≥6-<12 months, and ≥12 months between the positive test and the first dose (P = 0.7380).

### 3.4 Sex-specific differences in fatigue and quality of life scores

In the efficacy dataset group, there were 22 female participants. Overall, the female group showed significant improvements in all scores between the baseline and the 12-week time points. The mean total SF-36 score was 37.2 at baseline and increased to 56.5 at week 12 (P < 0.0001, Table 4).All scores within the SF-36 increased over the treatment period. The sum of the Chalder scale decreased from 26.6 at baseline to 15.9 at week 12 (P < 0.0001), and the decrease was significant for both the physical and mental fatigue scores. The improvement in mean general health was limited in this group and only observed at week 12.

**Table 4:** SF-36 and Chalder scores by sex.

	Mean (SD) of each item					p value of the difference vs baseline			
**Female (N = 22)**	Baseline	Week 2	Week 4	Week 8	Week 12	Week 2 vs. baseline	Week 4 vs. baseline	Week 8 vs. baseline	Week 12 vs. baseline
**Mean Total SF-36**	37.2 (15.6)	44.5 (16.8)	46.4 (17.4)	55.8 (20.4)	56.5 (23.1)	**0.0222**	**0.002**	**<0.0001**	**<0.0001**
Mean Physical functioning	58.6 (27.0)	64.1 (25.1)	65.6 (25.6)	66.9 (28.5)	71.0 (25.9)	0.1421	**0.0247**	**0.0224**	**0.0002**
Mean Role limitations due to physical health	9.1 (16.4)	25.0 (35.4)	23.4 (37.0)	34.4 (38.6)	45.0 (40.3)	0.0554	0.0552	**0.0036**	**0.0001**
Mean Role limitations due to emotional problems	16.7 (30.4)	27.3 (35.1)	41.7 (41.3)	79.2 (40.1)	55.6 (48.2)	0.2802	**0.0228**	**<0.0001**	**0.0006**
Mean Energy/fatigue	15.9 (12.1)	27.5 (17.8)	28.8 (16.8)	33.4 (20.8)	40.0 (26.3)	**0.0009**	**0.0001**	**<0.0001**	**<0.0001**
Mean Emotional well-being	50.2 (17.5)	53.8 (14.2)	57.0 (15.3)	69.5 (15.4)	67.7 (17.0)	0.2613	0.0626	**<0.0001**	**<0.0001**
Mean Social functioning	35.8 (23.2)	43.8 (23.4)	53.1 (22.6)	62.5 (22.4)	65.0 (26.8)	0.1186	**0.0008**	**<0.0001**	**<0.0001**
Mean Pain	51.0 (22.8)	61.4 (22.3)	63.6 (23.3)	74.7 (24.5)	73.2 (26.5)	**0.0139**	**0.0019**	**<0.0001**	**<0.0001**
Mean General Health	40.7 (22.3)	44.8 (22.3)	40.0 (22.6)	48.1 (23.1)	46.3 (23.1)	0.1883	0.6111	0.1088	**0.0487**
**Total Sum of Chalder Fatigue Scale**	26.1 (4.2)	20.1 (7.0)	18.4 (7.8)	16.7 (9.5)	15.9 (9.1)	**0.0003**	**<0.0001**	**<0.0001**	**<0.0001**
Sum of Chalder Fatigue Scale - Physical	16.9 (3.1)	13.2 (4.3)	12.4 (4.9)	11.1 (6.3)	10.7 (5.5)	**0.0011**	**0.0001**	**<0.0001**	**<0.0001**
Sum of Chalder Fatigue Scale - Mental	9.2 (1.8)	6.9 (3.1)	6.1 (3.4)	5.6 (3.6)	5.3 (4.0)	**0.0001**	**<0.0001**	**<0.0001**	**<0.0001**
**Male (N = 9)**	Baseline	Week 2	Week 4	Week 8	Week 12	Week 2 vs. baseline	Week 4 vs. baseline	Week 8 vs. baseline	Week 12 vs. baseline
**Mean Total SF-36**	34.7 (16.5)	35.1 (23.7)	36.8 (26.5)	39.6 (24.9)	43.8 (27.4)	0.0866	0.1846	**0.0338**	**0.0005**
Mean Physical functioning	53.1 (30.6)	52.5 (36.3)	52.8 (34.5)	58.3 (35.8)	58.8 (35.2)	0.1131	0.3593	**0.028**	**0.0075**
Mean Role limitations due to physical health	9.4 (12.9)	15.6 (35.2)	16.7 (33.1)	19.4 (39.1)	28.1 (38.8)	0.135	0.2226	0.1198	**0.0154**
Mean Role limitations due to emotional problems	29.2 (37.5)	20.8 (30.5)	29.6 (42.3)	25.9 (32.4)	50.0 (47.1)	0.55	0.9773	0.7661	0.0909
Mean Energy/fatigue	11.3 (8.3)	20.0 (16.0)	19.4 (21.7)	24.4 (24.4)	28.1 (26.2)	**0.0215**	0.0588	**0.0063**	**0.0017**
Mean Emotional well-being	50.5 (14.6)	49.5 (21.1)	45.8 (26.6)	52.4 (20.3)	54.0 (24.7)	0.5123	0.6262	0.427	0.1059
Mean Social functioning	37.5 (34.7)	37.5 (29.9)	43.1 (39.1)	43.1 (31.9)	40.6 (33.2)	0.2544	0.2136	0.2136	0.1608
Mean Pain	37.8 (30.2)	38.1 (30.1)	37.5 (33.2)	43.6 (28.5)	46.3 (29.3)	0.1831	0.8423	0.1922	0.0929
Mean General Health	35.0 (20.2)	29.4 (19.9)	36.7 (25.6)	34.4 (22.8)	35.6 (25.1)	0.5493	0.1825	0.493	0.0993
**Total Sum of Chalder Fatigue Scale**	25.4 (5.9)	24.8 (7.1)	22.4 (9.0)	21.8 (8.8)	20.1 (10.6)	0.0646	0.0062	**0.0018**	**<0.0001**
Sum of Chalder Fatigue Scale - Physical	17.4 (3.4)	16.5 (4.2)	15.0 (5.8)	13.9 (6.1)	12.9 (7.1)	0.0949	**0.0152**	**0.0013**	**0.0001**
Sum of Chalder Fatigue Scale - Mental	8.0 (2.6)	8.3 (3.2)	7.4 (3.4)	7.9 (3.1)	7.3 (3.8)	0.1681	**0.0231**	0.1702	**0.0011**

SD: standard deviation; SF-36: short form 36.

The cohort consisted of 9 men, and in this group a significant increase in the SF-36 scores from 34.7 at baseline to 43.8 at week 12 was detected (p = 0.0005, Table 4). However, there was no significant increase in scores related to role limitations due to emotional problems, emotional well-being, social functioning, pain, or general health, even though the scores increased for all scores in the treatment period. The Chalder Fatigue scale score for this group decreased from 25.4 at baseline to 20.1 at week 12 (P < 0.0001), with significant decreases in both the physical and mental fatigue scores. The differences in significant results between the female and male groups may be due to the small sample size of the male group.

### 3.5 Age-specific differences in fatigue and quality of life scores

In the intervention group, 11 participants were aged ≤39, 11 participants were aged >30-≤49, and 9 participants were aged above 49 (Table 5). The youngest age group did not experience significant improvements in physical functioning, emotional well-being, or pain levels as measured by the SF-36 after 12 weeks of treatment. The age group 39–49 years did not experience a significant improvement in role limitations due to emotional problems and general health, and in the age group above 49 years, no significant improvement was detected for role limitations due to emotional problems and general health.

**Table 5:** SF-36 and Chalder scores by age.

	Mean (SD) of each item					p value of the difference vs. baseline			
**Age<=39 (N = 11)**	Baseline	Week 2	Week 4	Week 8	Week 12	Week 2 vs. baseline	Week 4 vs. baseline	Week 8 vs. baseline	Week 12 vs. baseline
**Mean Total SF-36**	33.5 (18.9)	39.1 (25.1)	36.2(29.0)	43.2 (35.5)	40.8 (33.2)	0.057	0.0538	**0.0014**	**0.0008**
Mean Physical functioning	58.5 (35.5)	55.0 (36.7)	51.7(39.7)	47.5 (42.6)	46.9 (38.2)	0.8918	0.8963	0.5789	0.9765
Mean Role limitations due to physical health	10.0 (17.5)	31.8 (42.0)	33.3(50.0)	37.5 (51.8)	37.5 (51.8)	**0.024**	**0.0058**	**0.0054**	**0.0012**
Mean Role limitations due to emotional problems	16.7 (28.3)	21.2 (27.0)	40.7(46.5)	54.2 (46.9)	50.0 (53.5)	0.7068	0.06	**0.0062**	**0.0115**
Mean Energy/fatigue	12.0 (11.8)	23.6 (20.7)	22.2(25.5)	26.9 (33.8)	28.8 (37.2)	**0.0223**	**0.0321**	**0.0012**	**0.0007**
Mean Emotional well-being	44.4 (14.9)	46.2 (18.4)	37.8(20.2)	54.0 (24.2)	51.5 (22.2)	0.5653	0.5852	0.0733	0.067
Mean Social functioning	28.8 (32.8)	45.5 (34.6)	30.6(29.4)	50.0 (42.8)	39.1 (38.6)	**0.0147**	0.3666	**0.0048**	**0.0193**
Mean Pain	40.3 (24.5)	48.9 (32.6)	38.6 (30.6)	51.6 (36.9)	43.4 (32.6)	0.0841	0.5521	**0.0243**	0.0988
Mean General Health	29.5 (17.2)	34.5 (20.2)	27.2 (14.4)	41.3 (29.5)	35.0 (23.3)	**0.0385**	0.1594	**0.0003**	**0.0006**
**Total Sum of Chalder Fatigue Scale**	27.2 (4.8)	21.5 (9.5)	23.1 (10.0)	20.6 (13.6)	22.0 (11.9)	**0.0025**	**0.0061**	**0.0001**	**0.0005**
Sum of Chalder Fatigue Scale - Physical	17.9 (3.3)	14.1 (6.0)	14.9 (6.6)	12.8 (9.4)	14.3 (7.8)	**0.008**	**0.0102**	**0.0002**	**0.0015**
Sum of Chalder Fatigue Scale - Mental	9.3 (1.9)	7.4 (3.8)	8.2 (3.5)	7.9 (4.3)	7.8 (4.2)	**0.0016**	**0.0133**	**0.0004**	**0.0007**
**39<Age<=49 (N = 11)**	Baseline	Week 2	Week 4	Week 8	Week 12	Week 2 vs. baseline	Week 4 vs. baseline	Week 8 vs. baseline	Week 12 vs. baseline
**Mean Total SF-36**	32.2 (11.7)	36.9 (13.2)	43.2 (16.9)	49.7 (16.2)	51.6 (16.5)	0.2473	**0.0089**	**0.0002**	**<0.0001**
Mean Physical functioning	45.9 (17.3)	55.9 (18.4)	60.0 (20.8)	67.2 (24.4)	72.2 (17.7)	**0.0483**	**0.0064**	**0.0001**	**<0.0001**
Mean Role limitations due to physical health	2.3 (7.5)	9.1 (23.1)	16.7 (28.0)	19.4 (27.3)	30.6 (30.0)	0.4546	0.1157	0.0654	**0.0043**
Mean Role limitations due to emotional problems	21.2 (34.2)	24.2 (39.7)	29.6 (42.3)	66.7 (50.0)	51.9 (50.3)	0.8443	0.5642	**0.0074**	0.0603
Mean Energy/fatigue	10.0 (8.1)	18.2 (14.4)	23.9 (15.0)	25.0 (13.0)	29.4 (13.6)	0.055	**0.0012**	**0.0006**	**<0.0001**
Mean Emotional well-being	54.9 (18.7)	54.5 (15.4)	64.4 (16.5)	70.2 (17.3)	65.3 (19.9)	0.9287	0.0697	**0.0029**	**0.0453**
Mean Social functioning	33.0 (17.9)	33.0 (16.1)	56.9 (24.3)	54.2 (18.8)	61.1 (22.9)	1.000	**0.0003**	**0.001**	**0.0001**
Mean Pain	43.0 (25.2)	53.6 (25.3)	57.2 (26.3)	66.9 (26.9)	71.1 (22.3)	0.0636	**0.0044**	**<0.0001**	**<0.0001**
Mean General Health	38.2 (23.9)	36.4 (24.9)	38.3 (27.2)	38.3 (23.0)	38.3 (21.2)	0.6824	0.9672	0.9672	0.9672
**Total Sum of Chalder Fatigue Scale**	27.9 (3.4)	23.2 (6.7)	21.4 (6.9)	19.1 (8.6)	18.9 (6.9)	**0.041**	**0.0059**	**0.0004**	**0.0003**
Sum of Chalder Fatigue Scale - Physical	17.9 (2.8)	15.4 (4.0)	14.2 (4.2)	12.4 (5.5)	12.3 (3.8)	0.0903	**0.0139**	**0.0007**	**0.0006**
Sum of Chalder Fatigue Scale - Mental	10.0 (1.5)	7.8 (3.1)	7.2 (3.2)	6.7 (3.7)	6.6 (3.7)	**0.0203**	**0.0046**	**0.001**	**0.0007**
**Age>49 (N = 9)**	Baseline	Week 2	Week 4	Week 8	Week 12	Week 2 vs. baseline	Week 4 vs. baseline	Week 8 vs. baseline	Week 12 vs. baseline
**Mean Total SF-36**	45.3 (13.7)	53.1 (12.1)	51.2 (11.8)	57.0 (12.0)	67.9 (15.3)	0.1024	0.2478	**0.0378**	**0.0005**
Mean Physical functioning	69.4 (24.8)	76.3 (23.1)	74.3 (19.2)	76.3 (16.4)	85.0 (12.2)	0.2086	0.2977	0.2724	**0.0189**
Mean Role limitations due to physical health	16.7 (17.7)	28.1 (36.4)	10.7 (13.4)	31.3 (37.2)	54.2 (36.8)	0.3339	0.6582	0.2876	**0.0200**
Mean Role limitations due to emotional problems	22.2 (37.3)	33.3 (35.6)	42.9 (37.1)	58.3 (42.7)	61.1 (39.0)	0.5492	0.2951	0.0598	0.0551
Mean Energy/fatigue	23.3 (10.0)	38.1 (8.8)	31.4 (13.8)	39.4 (14.0)	55.0 (14.8)	**0.0053**	0.0905	**0.0031**	**<0.0001**
Mean Emotional well-being	51.1 (15.3)	59.0 (11.3)	57.7 (13.0)	65.0 (11.5)	74.7 (12.3)	0.0667	0.2121	**0.0092**	**<0.0001**
Mean Social functioning	48.6 (24.6)	50.0 (16.4)	64.3 (24.4)	62.5 (14.9)	72.9 (20.0)	0.6018	0.0635	0.1102	**0.0029**
Mean Pain	61.1 (22.8)	65.9 (15.1)	70.4 (25.0)	71.6 (23.8)	80.0 (23.9)	0.2203	0.1562	0.1348	**0.0388**
Mean General Health	51.1 (19.0)	55.0 (17.3)	54.3 (20.5)	50.6 (17.8)	59.2 (23.8)	0.076	0.3299	0.5512	0.1347
**Total Sum of Chalder Fatigue Scale**	22.0 (3.4)	18.6 (2.3)	13.7 (3.9)	15.8 (4.5)	9.0 (3.8)	0.0576	**0.0001**	**0.0014**	**<0.0001**
Sum of Chalder Fatigue Scale - Physical	14.9 (2.3)	12.4 (1.4)	10.1 (3.4)	11.0 (3.1)	6.3 (2.1)	**0.0398**	**0.0008**	**0.0034**	**<0.0001**
Sum of Chalder Fatigue Scale - Mental	7.1 (1.7)	6.3 (1.9)	3.6 (1.0)	4.8 (2.0)	2.7 (2.0)	0.245	**0.0002**	**0.0053**	**<0.0001**

SD: standard deviation; SF-36: short form 36.

Survey scores corrected for age and sex.

A linear mixed model adjusted for age and sex was used to test the change of each health item across time. The coefficients presented in Table 6 show the change of each health item across time (using the baseline as the reference group) after adjusting for age and sex. In this model, significant improvements in all scores were detected between baseline and week 12 (Table 6).

**Table 6:** Linear mixed model for SF-36 and Chalder scores adjusted for age and gender.

	Week 2 vs. baseline		Week 4 vs. baseline		Week 8 vs. baseline		Week 12 vs. baseline	
	Coefficient	p value	Coefficient	p value	Coefficient	p value	Coefficient	p value
**Mean Total SF-36**	6.95	**0.0058**	9.24	**0.0007**	15.15	**<0.0001**	19.43	**<0.0001**
Mean Physical functioning	5.78	**0.0449**	7.47	**0.0160**	9.56	**0.0022**	14.95	**<0.0001**
Mean Role limitations due to physical health	14.70	**0.0200**	15.74	**0.0193**	22.79	**0.0008**	34.48	**<0.0001**
Mean Role limitations due to emotional problems	5.64	0.5080	18.24	**0.0457**	41.08	**<0.0001**	36.22	**0.0002**
Mean Energy/fatigue	11.92	**0.0001**	13.41	**<0.0001**	18.49	**<0.0001**	24.56	**<0.0001**
Mean Emotional well-being	3.58	0.2002	3.78	0.2052	12.68	**<0.0001**	14.23	**<0.0001**
Mean Social functioning	7.61	0.0677	16.12	**0.0004**	19.98	**<0.0001**	25.36	**<0.0001**
Mean Pain	9.59	**0.0056**	10.55	**0.0044**	17.98	**<0.0001**	19.94	**<0.0001**
Mean General Health	3.53	0.1399	2.88	0.2596	4.41	0.0855	6.72	**0.0115**
**Total Sum of Chalder Fatigue Scale**	−5.10	**<0.0001**	−7.06	**<0.0001**	−8.51	**<0.0001**	−9.99	**<0.0001**
Sum of Chalder Fatigue Scale - Physical	−3.19	**0.0002**	−4.38	**<0.0001**	−5.62	**<0.0001**	−6.49	**<0.0001**
Sum of Chalder Fatigue Scale - Mental	−1.90	**0.0001**	−2.66	**<0.0001**	−2.87	**<0.0001**	−3.48	**<0.0001**

SF-36: short form 36.

### 3.6 Clinically responsive participants

**Fig. 2:**
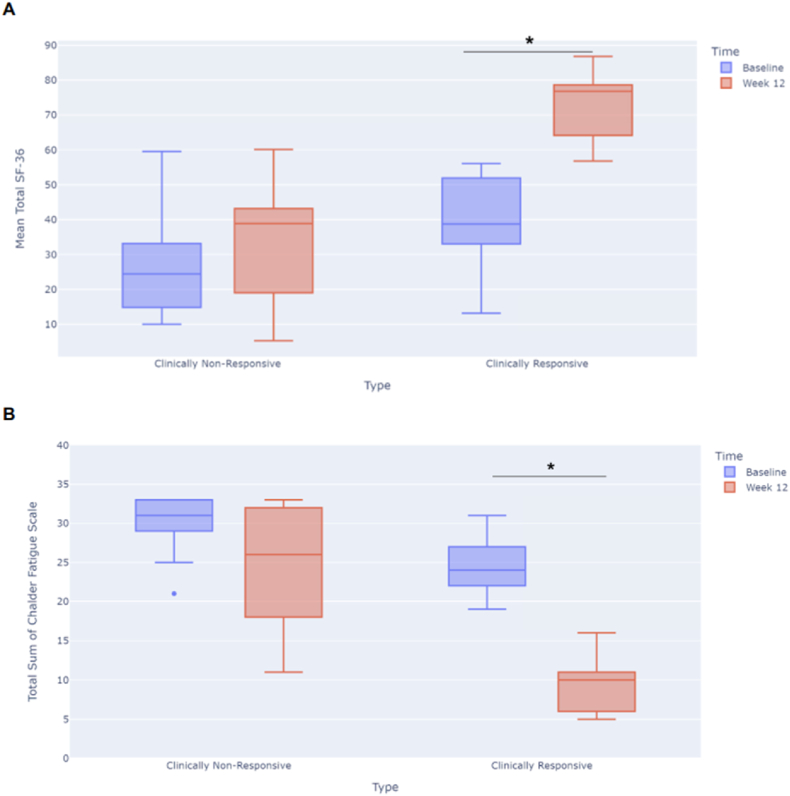
Boxplots depicting clinically responsive and non-responsive participants. Clinically responsive was defined as those participants with over 20 % of improvements in SF-36 scores at week 12 from baseline and with a week-12 score of ≥55. A) SF-36 scores at baseline and week 12, B) Chalder Fatigue scores at baseline and week 12. *P < 0.001.

Even though improvements were detected in both the SF-36 and Chalder scores for the total study population, there was variability in individual responses. To assess whether a specific subgroup could be defined as responders to the intervention, we defined those with over 20 % of improvements in SF-36 scores at week 12 from baseline and with a week-12 score of ≥55 as clinically responsive. A total of 11 participants fit these criteria. Fig. 2A shows a boxplot depicting the mean total SF-36 scores at baseline and week 12 for those who were considered clinically responsive (N = 11) and non-responsive (N = 12). Clinically responsive participants reported a significant increase in SF-36 scores (P < 0.0001), while those who were clinically non-responsive did not significantly improve in mean SF-36 scores 12 weeks after the start of treatment (P = 0.2656). When assessing the Chalder scores for these two groups (Fig. 2B), we also found a significant improvement in fatigue scores for the clinically responsive group (P < 0.0001), while the non-responsive group had limited improvements in fatigue scores (P = 0.0294). We did not detect a correlation between the length of time between the positive COVID-19 test and first dose of study drug and whether a participant was “clinically responsive” (p = 0.8372).

## 4 Discussion

In this study, we assessed the effects of LDN and NAD + supplementation on persistent moderate/severe fatigue symptoms after COVID-19. Case definitions for long COVID vary, and at the time of the initiation of this study, no biomarkers were defined to use as selection criteria for patients. We included those patients with Chalder fatigue survey scores of >9 in our study to select patients with moderate/severe fatigue indicative of post-viral fatigue syndrome. Patients who had ongoing fatigue symptoms after their acute infection before entering the study noted improvements in self-reported quality of life and fatigue survey scales. We included patients who were actively seeking out treatment for fatigue symptoms induced by COVID-19, which had lasted on average over six months before entering the study. Unfortunately, due to the accrual issues in this trial, we changed the format of this study to an observational study and were unable to directly compare the treatment to a control group. However, out of the participants who received LDN and NAD+ and completed their 12-week assessments, approximately half could be described as responders (those with ≥20 % improvement from baseline score and having a Week 12 value of >55 for SF-36 scores), suggesting a subgroup of patients who have long-term fatigue symptoms after COVID-19 might benefit from these treatments. We did not detect any significant differences between responders and non-responders in terms of age, sex, or time between the positive COVID-19 test and the start of treatment. Therefore, randomized controlled studies in various subpopulations will be needed to assess specific characteristics of this subgroup to optimize treatment and allow for the selection of patients who might benefit most from this treatment.

LDN has previously been suggested to be safe for use in patients diagnosed with multiple sclerosis, Crohn's disease, and fibromyalgia, with limited adverse events reported (Patten et al., 2018). This study confirms these data, and adverse events detected in this study were generally mild and were previously noted for LDN. Furthermore, most adverse events occurred only in the first weeks of treatment and could be managed with dose adjustments. In our study with a 12-week follow-up, we aimed to build up the dosing for LDN relatively fast to reach the therapeutic dose as quickly as possible. In clinical practice, the dose is usually titrated over several weeks to lower adverse events. In a previous study among 38 individuals with potential long COVID treated with LDN at 1–3 mg/day, two patients experienced adverse events that were the reason for cessation of therapy (fatigue and diarrhea) (O'Kelly et al., 2022). In our study, four patients withdrew due to expected adverse events from LDN, of whom three withdrew within the first two weeks of treatment.

Limited data are available on the safety of the NAD + iontophoresis patches. In this study, nine participants experienced skin irritation. However, it was considered mild in all cases, and only one participant wished to treat it with a topical corticosteroid cream. Skin irritation can also be prevented or managed by rotating sites to apply the patch.

The Chalder fatigue scale has previously been used in ME/CFS research. In a study comparing scores between people with ME/CFS and a healthy population, scores of 29 could discriminate between CFS and community samples in 96 % of cases (Cella and Chalder, 2010). The mean CFS score was 24.4 (SD: 5.8) and for the community was 14.2 (SD: 4.6). The baseline mean score of our cohort was 25.9 (SD: 4.6), suggesting similar fatigue levels for a large proportion of the participants in this study at baseline as patients with ME/CFS. This score decreased to 17.4 (SD: 9.7) at week 12 after treatment, and the responder subgroup of patients had lowered fatigue scores that were similar to the previously mentioned community samples.

A study for LDN in patients with long COVID also showed improvement in various factors after two months of therapy, including limitations in activities of daily living, energy, pain, concentration, sleep disturbance, and recovery from COVID-19. The largest improvement in that study was found to be pain-related (O'Kelly et al., 2022). We also detected significant improvements in scores for pain after two and three months of treatment (p < 0.0001), but only detected this effect in women. However, our study sample of men was small and larger studies will be needed to determine whether there is a real sex difference in pain reduction induced by LDN.

Supplementation of NAD + has been suggested for COVID-19 in particular for older patients as aging decreases the NAD + concentration in the body (Zheng et al., 2022). It has also been suggested as a treatment for ME/CFS (Castro-Marrero et al., 2021), in which supplementation of NADH and co-enzyme Q10 was shown to decrease fatigue symptoms and improve quality of life, similar to our study.

Given the lack of a comparable control group, the improvements in quality of life and fatigue scores detected in this study could be attributed partially to the placebo-effect or natural improvements in disease state over time. However, given that most of the patients signing up for our study reported having attempted other treatments without benefit and having had symptoms for an average of 223 days, our data suggest a subgroup of patients might benefit from LDN and NAD + treatment. A recent study in England showed that of those with persistent symptoms at 12 weeks after infection, 69 % still had symptoms after a year, suggesting only 31 % naturally recovered (Atchison et al., 2023). Of the 20 patients receiving treatment answering this question in our final survey, 15 responded with a desire to continue treatment, of whom 4 wanted to continue NAD + alone, and 1 wanted to continue LDN alone. Another four subjects considered the cost of NAD + to be prohibitive in continuing the regimen. Therefore, evaluations of more cost-efficient ways to provide NAD + treatment are warranted.

Furthermore, the establishment of the most appropriate dosing and duration of LDN treatment will have to be established. Here, we elected to test one dose for all patients. However, experience with LDN in other patient populations has shown that dosing might need to be personalized to improve outcomes and limit adverse events, both lower and higher doses might be needed in individuals, as in our clinical practice we have detected some patients requiring doses up to 6 mg/day to benefit from the therapy. Additionally, the duration of treatment may also affect efficacy. We chose 12 weeks as a reasonable timeframe to see clinical responses. However, it is possible a subset of patients may benefit from longer treatment.

A major limitation of our study is that we were not able to include a control arm due to accrual issues. As the tested treatments were available for participants outside of this trial, most chose to not participate in a trial in which they could receive a placebo. Therefore, we had to change the format to an observational open-label study, which comes with various limitations. This study design and the fact that participants were actively seeking out treatment may have confounded our data. Another limitation of our study is that we did not measure any objective measures for health improvements. Fatigue and pain levels are difficult to measure outside of subjective surveys. As this study was an open-label, single-arm investigation without a control group, a formal power analysis for between-group comparisons was deemed unnecessary. The sample size was therefore determined based on feasibility and resources, with the primary focus on characterizing outcomes within the treated group.

In conclusion, our study showed that treatment with LDN and NAD + may be beneficial in those with long-term fatigue symptoms resulting from COVID-19. Further studies will be needed to establish what subgroups of patients benefit most.

## Role of funding source

The research was funded by AgelessRx.

## CRediT authorship contribution statement

**Anar Isman:** Conceptualization, Supervision. **Andy Nyquist:** Data curation, Investigation, Project administration, Supervision, Writing – review & editing. **Bailey Strecker:** Project administration. **Girish Harinath:** Writing – review & editing. **Virginia Lee:** Project administration, Resources. **Xingyu Zhang:** Data curation, Formal analysis, Visualization. **Sajad Zalzala:** Conceptualization, Investigation, Methodology, Supervision, Writing – review & editing.

## Declaration of competing interest

The authors declare the following financial interests/personal relationships which may be considered as potential competing interests:

AgelessRx is a longevity-focused telemedicine platform that is the sponsor of several clinical trials, including interventional and observational efforts for use of repurposed gerotherapeutics. AI, AN, BS, GH, VL, SZ are employees of AgelessRx.

## Data Availability

Data will be made available on request.
