# Supplementary figures and images for: Low-dose naltrexone and NAD+ for the treatment of patients with persistent fatigue symptoms after COVID-19
Source: Brain Behav Immun Health. 2024 Feb 1;36:100733. doi: 10.1016/j.bbih.2024.100733 (PMC10862402; doi:10.1016/j.bbih.2024.100733)

**
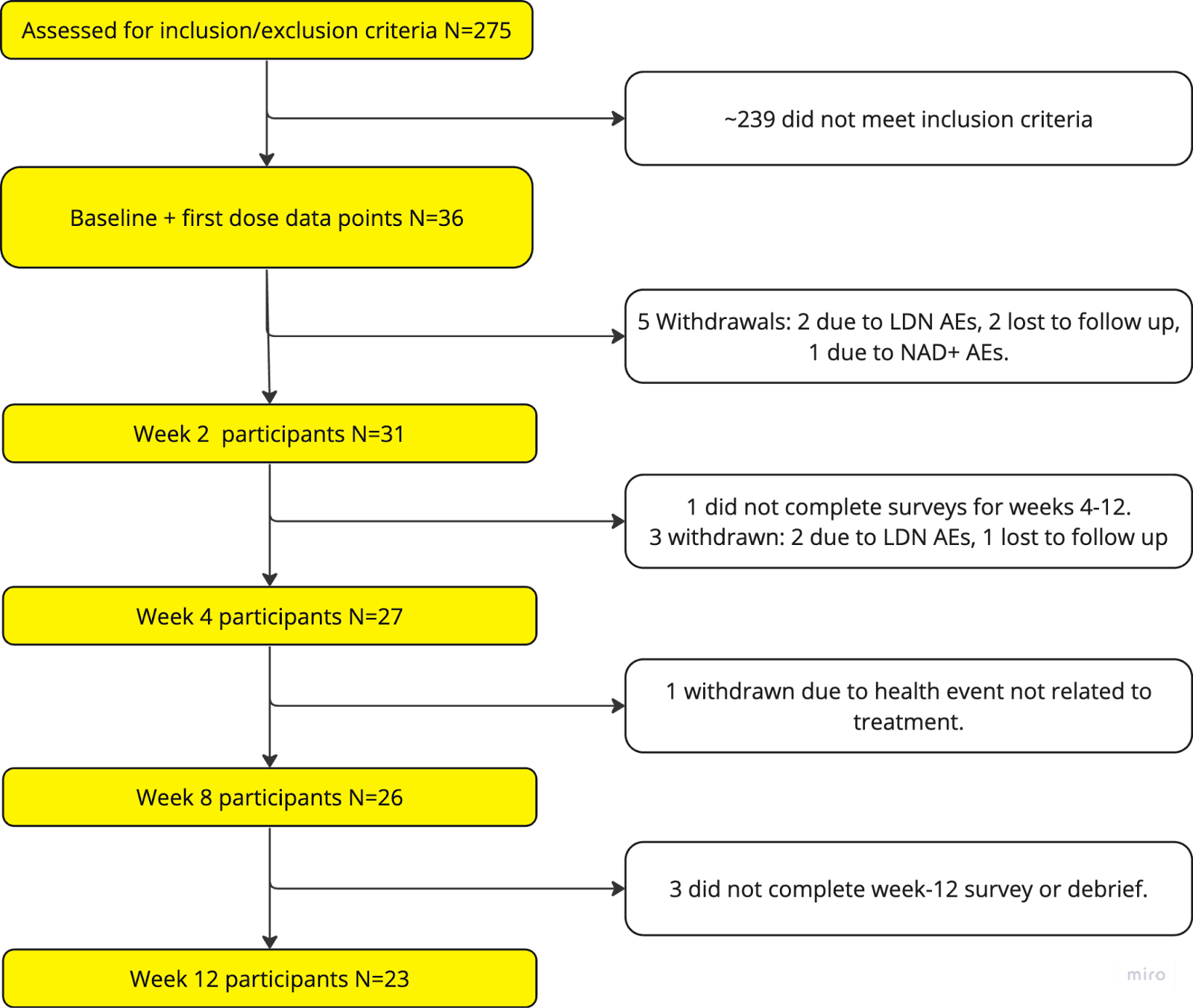
**

**Supplementary Figure S1: Study flowchart***AE: adverse events; LDN: low-dose naltrexone.*

Supplement: Multimedia component 1 [file mmc1.docx]
